# Supplementary material for: Romantic Attraction and Substance Use in 15-Year-Old Adolescents from Eight European Countries
Source: Int J Environ Res Public Health. 2019 Aug 23;16(17):3063. doi: 10.3390/ijerph16173063 (PMC6747452; doi:10.3390/ijerph16173063)
Supplement: Supplementary file 1 [file ijerph-16-03063-s001.pdf]

Table S1 (supplement). Crude and adjusted odds for cigarette smoking in the last 30 days, overall and by gender (n = 13,504)

	**Univariate models**			**Multivariate model (overall)**			**Multivariate model stratified for sex**					
							**Boys (n = 6693)**			**Girls (n = 6811)**		
	**COR [1]**	***p***	**(95% CI)**	**AOR [2]**	***p***	**(95% CI)**	**AOR**	***p***	**(95% CI)**	**AOR**	***p***	**(95% CI)**
**Love**												
Opposite-sex love	1			1			1			1		
Same-sex love	**2.00**	< .001	(1.48–2.69)	**1.85**	< .001	(1.34–2.55)	**2.36**	< .001	(1.46–3.82)	**1.57**	< .001	(1.02–2.41)
Both-sex love	**2.28**	< .001	(1.74–2.99)	**2.31**	< .001	(1.71–3.13)	**2.80**	< .001	(1.65–4.75)	**2.10**	< .001	(1.44–3.04)
Not in love	**0.47**	< .001	(0.40–0.56)	**0.44**	< .001	(0.37–0.52)	**0.53**	< .001	(0.41–0.70)	**0.38**	< .001	(0.30–0.48)
Not responding	1.08	.599	(0.81–1.43)	0.89	.450	(0.67–1.20)	1.12	.584	(0.75–1.65)	0.68	.093	(0.44–1.07)
**Country**												
Belgium (French)	1			1			1			1		
Bulgaria	**1.64**	< .001	(1.40–1.93)	**1.58**	< .001	(1.35–1.87)	**1.31**	.024	(1.04–1.66)	**1.89**	< .001	(1.51–2.37)
Switzerland	0.86	.069	(0.72–1.01)	0.85	.068	(0.72–1.01)	0.96	.766	(0.75–1.24)	**0.76**	.024	(0.60–0.96)
England	**0.55**	< .001	(0.46–0.67)	**0.67**	< .001	(0.55–0.82)	**0.50**	< .001	(0.36–0.68)	0.85	.240	(0.66–1.11)
France	**1.46**	< .001	(1.15–1.60)	**1.38**	< .001	(1.17–1.63)	**1.34**	.022	(1.04–1.71)	**1.42**	.003	(1.13–1.78)
Hungary	**1.65**	< .001	(1.38–1.96)	**1.69**	< .001	(1.42–2.02)	**1.57**	.001	(1.21–2.04)	**1.79**	< .001	(1.41–2.28)
Iceland	**0.21**	< .001	(0.17–0.25)	**0.19**	< .001	(0.16–0.23)	**0.18**	< .001	(0.14–0.25)	**0.20**	< .001	(0.15–0.26)
North Macedonia	**0.71**	< .001	(0.59–0.86)	**0.73**	< .001	(0.61–0.88)	**0.75**	.037	(0.57–0.99)	**0.71**	.01	(0.55–0.92)
**Relative FAS**												
Lowest 20 percent	1			1			1			1		
Medium 60 percent	1.08	.194	(0.96–1.21)	0.98	.786	(0.87–1.11)	1.07	.435	(0.90–1.28)	0.92	.323	(0.76–1.09)
Highest 20 percent	**1.32**	< .001	(1.15–1.52)	**1.27**	.001	(1.10–1.47)	**1.28**	.026	(1.03–1.59)	**1.26**	.022	(1.03–1.54)
**Sex**												
Boy	1			1								
Girl	**1.21**	< .001	(1.11–1.33)	**1.26**	< .001	(1.15–1.38)						

[1] COR: Crude odds ratios. [2] AOR: Odds ratios adjusted for region, gender, and relative family affluence. Boldface indicates statistically significant differences in ($p < .05$) odds for cigarette smoking in the given group, as compared to the reference group.

Table S2 (supplement). Crude and adjusted odds for alcohol consumption in the last 30 days, overall and by gender (n = 13,440)

	**Univariate models**			**Multivariate model (overall)**			**Multivariate model stratified for sex**					
							**Boys (n = 6656)**			**Girls (n = 6784)**		
	**COR** [1]	***p***	**(95% CI)**	**AOR** [2]	***p***	**(95% CI)**	**AOR**	***p***	**(95% CI)**	**AOR**	***p***	**(95% CI)**
**Love**												
Opposite-sex love	1			1			1			1		
Same-sex love	1.27	.093	(0.96–1.66)	1.20	.230	(0.89–1.63)	**1.66**	.036	(1.03–2.66)	0.97	.880	(0.64–1.46)
Both-sex love	**1.67**	< .001	(1.29–2.15)	**1.80**	< .001	(1.33–2.43)	1.08	.784	(0.64–1.81)	**2.15**	< .001	(1.50–3.08)
Not in love	**0.67**	< .001	(0.60–0.75)	**0.52**	< .001	(0.46–0.59)	**0.56**	< .001	(0.47–0.67)	**0.49**	< .001	(0.42–0.58)
Not responding	**0.66**	.001	(0.52–0.84)	**0.48**	< .001	(0.38–0.62)	**0.57**	.001	(0.41–0.79)	**0.37**	< .001	(0.26–0.56)
**Country**												
Belgium (French)	1			1			1			1		
Bulgaria	**1.38**	< .001	(1.20–1.58)	**1.32**	< .001	(1.14–1.51)	**1.36**	.002	(1.12–1.66)	**1.26**	.023	(1.03–1.54)
Switzerland	**0.78**	< .001	(0.68–0.89)	**0.76**	< .001	(0.66–0.87)	0.84	.073	(0.69–1.02)	**0.69**	< .001	(0.57–0.83)
England	**0.81**	.004	(0.71–0.93)	0.98	.735	(0.84–1.13)	**0.78**	.022	(0.64–0.97)	1.22	.057	(0.99–1.50)
France	**0.75**	< .001	(0.65–0.86)	**0.74**	< .001	(0.64–0.85)	**0.81**	.041	(0.66–0.99)	**0.67**	< .001	(0.54–0.81)
Hungary	**1.32**	< .001	(1.14–1.54)	**1.38**	< .001	(1.18–1.61)	**1.45**	.001	(1.16–1.82)	**1.30**	.015	(1.05–1.61)
Iceland	**0.14**	< .001	(0.12–0.16)	**0.13**	< .001	(0.11–0.15)	**0.11**	< .001	(0.09–0.14)	**0.14**	< .001	(0.11–0.17)
North Macedonia	**0.45**	< .001	(0.39–0.52)	**0.46**	< .001	(0.40–0.54)	**0.51**	< .001	(0.41–0.63)	**0.41**	< .001	(0.33–0.51)
**Relative FAS**												
Lowest 20 percent	1			1			1			1		
Medium 60 percent	**1.49**	< .001	(1.35–1.63)	**1.34**	< .001	(1.22–1.49)	**1.34**	< .001	(1.17–1.54)	**1.37**	< .001	(1.19–1.59)
Highest 20 percent	**1.75**	< .001	(1.56–1.96)	**1.81**	< .001	(1.60–2.04)	**1.75**	< .001	(1.47–2.08)	**1.91**	< .001	(1.61–2.28)
**Sex**												
Boy	1			1								
Girl	**0.91**	.007	(0.85–0.98)	**0.91**	.012	(0.84–0.98)						

[1] COR: Crude odds ratios. [2] AOR: Odds ratios adjusted for region, gender, and relative family affluence. Boldface indicates statistically significant differences in ($p < .05$) odds for alcohol consumption in the given group, as compared to the reference group.

Table S3 (supplement). Crude and adjusted odds for drunkenness in the last 30 days, overall and by gender (n = 13,471)

	Univariate models			Multivariate model (overall)			Multivariate model stratified for sex					
							Boys (n = 6693)			Girls (n = 6811)		
	**COR [1]**	***p***	**(95% CI)**	**AOR [2]**	***p***	**(95% CI)**	**AOR**	***p***	**(95% CI)**	**AOR**	***p***	**(95% CI)**
**Love**												
Opposite-sex love	1			1			1			1		
Same-sex love	**1.92**	< .001	(1.39–2.67)	**1.81**	.001	(1.28–2.55)	**2.42**	< .001	(1.48–3.96)	1.38	.204	(0.84–2.28)
Both-sex love	**2.20**	< .001	(1.63–2.96)	**2.19**	< .001	(1.59–3.02)	**1.93**	.016	(1.13–3.31)	**2.25**	< .001	(1.51–3.34)
Not in love	**0.65**	< .001	(0.55–0.77)	**0.51**	< .001	(0.43–0.61)	**0.55**	< .001	(0.42–0.72)	**0.47**	< .001	(0.37–0.60)
Not responding	0.98	.879	(0.70–1.36)	**0.68**	.021	(0.48–0.94)	0.71	.120	(0.46–1.09)	0.63	.090	(0.37–1.07)
**Country**												
Belgium (French)	1			1			1			1		
Bulgaria	**1.85**	< .001	(1.55–2.21)	**1.75**	< .001	(1.47–2.09)	**2.01**	< .001	(1.58–2.56)	**1.44**	.008	(1.10–1.87)
Switzerland	**0.72**	.001	(0.59–0.88)	**0.71**	.001	(0.58–0.87)	0.76	.058	(0.58–1.01)	**0.66**	.005	(0.49–0.88)
England	**1.27**	.014	(1.05–1.53)	**1.51**	< .001	(1.24–1.84)	1.06	.694	(0.79–1.42)	**2.10**	< .001	(1.61–2.75)
France	**0.68**	< .001	(0.55–0.84)	**0.67**	< .001	(0.54–0.83)	**0.62**	.002	(0.46–0.84)	**0.74**	.042	(0.55–0.99)
Hungary	**2.05**	< .001	(1.70–2.48)	**2.11**	< .001	(1.75–2.56)	**2.09**	< .001	(1.59–2.75)	**2.13**	< .001	(1.63–2.79)
Iceland	**0.22**	< .001	(0.18–0.28)	**0.21**	< .001	(0.17–0.27)	**0.19**	< .001	(0.14–0.26)	**0.23**	< .001	(0.17–0.32)
North Macedonia	**0.65**	< .001	(0.52–0.81)	**0.66**	< .001	(0.53–0.83)	0.76	.065	(0.56–1.02)	**0.56**	.001	(0.40–0.76)
**Relative FAS**												
Lowest 20 percent	1			1			1			1		
Medium 60 percent	**1.29**	< .001	(1.13–1.48)	**1.18**	.020	(1.03–1.36)	1.18	.082	(0.98–1.44)	1.18	.112	(0.96–1.45)
Highest 20 percent	**1.65**	< .001	(1.40–1.93)	**1.64**	< .001	(1.39–1.94)	**1.68**	< .001	(1.34–2.12)	**1.63**	< .001	(1.28–2.07)
**Sex**												
Boy	1			1								
Girl	**0.88**	.014	(0.79–0.98)	**0.89**	.021	(0.80–0.98)						

[1] COR: Crude odds ratios. [2] AOR: Odds ratios adjusted for region, gender, and relative family affluence. Boldface indicates statistically significant differences in (p < .05) odds for drunkenness in the given group, as compared to the reference group.

Table S4 (supplement). Crude and adjusted odds for cannabis use in the last 30 days, overall and by gender (n = 12,109)

	Univariate models			Multivariate model (overall)			Multivariate model stratified for sex					
							Boys (n = 5999)			Girls (n = 6110)		
	**COR[1]**	***p***	**(95% CI)**	**AOR[2]**	***p***	**(95% CI)**	**AOR**	***p***	**(95% CI)**	**AOR**	***p***	**(95% CI)**
**Love**												
Opposite-sex love	1			1			1			1		
Same-sex love	**2.21**	< .001	(1.48–3.31)	**2.16**	.001	(1.39–3.36)	**2.90**	.001	(1.57–5.38)	1.62	.142	(0.85–3.09)
Both-sex love	**3.19**	< .001	(2.30–4.44)	**3.57**	< .001	(2.48–5.13)	**4.12**	< .001	(2.33–7.30)	**3.20**	< .001	(1.99–5.15)
Not in love	**0.68**	.001	(0.54–0.85)	**0.62**	< .001	(0.48–0.79)	**0.71**	.048	(0.51–1.00)	**0.53**	.001	(0.36–0.77)
Not responding	1.18	.581	(0.66–2.10)	1.05	.979	(0.57–1.93)	1.10	.821	(0.50–2.40)	1.01	.990	(0.38–2.65)
**Country**												
Belgium (French)	1			1			1			1		
Bulgaria	1.02	.920	(0.75–1.39)	0.96	.814	(0.70–1.32)	0.95	.805	(0.61–1.48)	0.97	.895	(0.61–1.53)
Switzerland	1.23	.181	(0.91–1.67)	1.28	.115	(0.94–1.75)	1.43	.106	(0.93–2.21)	1.11	.645	(0.71–1.73)
England	0.78	.131	(0.56–1.08)	0.89	.513	(0.64–1.25)	0.80	.370	(0.50–1.30)	1.01	.954	(0.63–1.63)
France	**1.47**	.014	(1.08–1.99)	**1.50**	.010	(1.10–2.05)	1.50	.072	(0.96–2.33)	1.51	.064	(0.98–2.33)
Hungary	**0.43**	< .001	(0.29–0.64)	**0.44**	< .001	(0.30–0.65)	**0.43**	.003	(0.25–0.75)	**0.45**	.005	(0.26–0.79)
Iceland	**0.20**	< .001	(0.14–0.29)	**0.19**	< .001	(0.13–0.28)	**0.19**	< .001	(0.11–0.31)	**0.20**	< .001	(0.12–0.33)
North Macedonia	**0.17**	< .001	(0.11–0.28)	**0.17**	< .001	(0.11–0.28)	**0.20**	< .001	(0.11–0.38)	**0.14**	< .001	(0.06–0.30)
**Relative FAS**												
Lowest 20 percent	1			1			1			1		
Medium 60 percent	1.06	.529	(0.88–1.27)	0.96	.694	(0.80–1.16)	1.00	.977	(0.78–1.27)	0.94	.690	(0.70–1.26)
Highest 20 percent	**1.51**	< .001	(1.22–1.85)	**1.57**	< .001	(1.27–1.95)	**1.50**	.007	(1.12–2.00)	**1.67**	.002	(1.21–2.31)
**Sex**												
Boy	1			1								
Girl	**0.75**	< .001	(0.66–0.86)	**0.72**	< .001	(0.63–0.84)						

[1] COR: Crude odds ratios. [2] AOR: Odds ratios adjusted for region, gender, and relative family affluence. Boldface indicates statistically significant differences in (p < .05) odds for drunkenness in the given group, as compared to the reference group.

Table S5 (supplement). Crude and adjusted odds for multiple substance use in the last 30 days, overall and by gender (n = 13,580)

	Univariate models			Multivariate model (overall)			Multivariate model stratified for sex					
							Boys (n = 6848)			Girls (n = 6732)		
	**COR** [1]	***p***	**(95% CI)**	**AOR** [2]	***p***	**(95% CI)**	**AOR**	***p***	**(95% CI)**	**AOR**	***p***	**(95% CI)**
**Love**												
Opposite-sex love	1			1			1			1		
Same-sex love	**1.79**	< .001	(1.30–2.46)	**1.68**	.003	(1.20–2.35)	**2.12**	.002	(1.33–3.64)	1.39	.161	(0.88–2.20)
Both-sex love	**2.34**	< .001	(1.77–3.09)	**2.43**	< .001	(1.79–3.31)	**2.78**	< .001	(1.64–4.70)	**2.24**	< .001	(1.54–3.27)
Not in love	**0.47**	< .001	(0.39–0.56)	**0.44**	< .001	(0.36–0.53)	**0.53**	< .001	(0.40–0.70)	**0.38**	< .001	(0.29–0.49)
Not responding	0.84	.291	(0.60–1.16)	0.74	.081	(0.53–1.03)	0.90	.651	(0.58–1.41)	**0.59**	.044	(0.35–0.99)
**Country**												
Belgium (French)	1			1			1			1		
Bulgaria	**1.57**	< .001	(1.33–1.86)	**1.51**	< .001	(1.28–1.79)	**1.36**	.015	(1.06–1.73)	**1.67**	< .001	(1.32–2.11)
Switzerland	1.07	.433	(0.90–1.27)	1.07	.451	(0.90–1.27)	1.28	.052	(1.00–1.65)	0.89	.341	(0.69–1.13)
England	**0.65**	< .001	(0.53–0.79)	**0.80**	.029	(0.65–0.98)	**0.60**	.002	(0.44–0.83)	1.00	.999	(0.76–1.31)
France	**1.31**	.002	(1.10–1.56)	**1.33**	.002	(1.11–1.58)	**1.40**	.011	(1.08–1.81)	1.25	.068	(0.98–1.60)
Hungary	**1.55**	< .001	(1.29–1.87)	**1.60**	< .001	(1.35–1.96)	**1.52**	.003	(1.16–2.00)	**1.66**	< .001	(1.30–2.14)
Iceland	**0.19**	< .001	(0.16–0.24)	**0.18**	< .001	(0.15–0.23)	**0.17**	< .001	(0.12–0.24)	**0.19**	< .001	(0.14–0.25)
North Macedonia	**0.45**	< .001	(0.36–0.56)	**0.46**	< .001	(0.37–0.58)	**0.45**	< .001	(0.33–0.63)	**0.47**	< .001	(0.34–0.63)
**Relative FAS**												
Lowest 20 percent	1			1			1			1		
Medium 60 percent	**1.18**	.010	(1.04–1.34)	1.06	.355	(0.93–1.21)	1.08	.431	(0.90–1.30)	1.08	.410	(0.90–1.31)
Highest 20 percent	**1.54**	< .001	(1.32–1.78)	**1.50**	< .001	(1.29–1.76)	**1.44**	.001	(1.15–1.81)	**1.60**	< .001	(1.29–1.98)
**Sex**												
Boy	1			1								
Girl	1.10	.051	(1.00–1.21)	**1.12**	.024	(1.01–1.23)						

[1] COR: Crude odds ratios. [2] AOR: Odds ratios adjusted for region, gender, and relative family affluence. Boldface indicates statistically significant differences in ($p < .05$) odds for drunkenness in the given group, as compared to the reference group.
